# Using Bacteriophages as a Trojan Horse to the Killing of Dual-Species Biofilm Formed by *Pseudomonas aeruginosa* and Methicillin Resistant *Staphylococcus aureus*

**DOI:** 10.3389/fmicb.2020.00695

**Published:** 2020-04-15

**Authors:** Tamta Tkhilaishvili, Lei Wang, Carsten Perka, Andrej Trampuz, Mercedes Gonzalez Moreno

**Affiliations:** ^1^Centre for Musculoskeletal Surgery, Charité – Universitätsmedizin Berlin, Corporate Member of Freie Universität Berlin, Humboldt-Universität zu Berlin, and Berlin Institute of Health, Berlin, Germany; ^2^BIH Center for Regenerative Therapies, Charité – Universitätsmedizin Berlin, Berlin, Germany; ^3^Berlin-Brandenburg School for Regenerative Therapies, Charité – Universitätsmedizin Berlin, Berlin, Germany

**Keywords:** *Pseudomonas aeruginosa*, methicillin-resistant *Staphylococcus aureus*, biofilm-associated infection, dual-species biofilm, antibiotic-bacteriophage combination, bacteriophages, isothermal microcalorimetry, scanning electron microscopy

## Abstract

*Pseudomonas aeruginosa* and *Staphylococcus aureus* are pathogens able to colonize surfaces and form together a mixed biofilm. Dual-species biofilms are significantly more resistant to antimicrobials than a monomicrobial community, leading to treatment failure. Due to their rapid bactericidal activity, the self-amplification ability and the biofilm degrading properties, bacteriophages represent a promising therapeutic option in fighting biofilm-related infections. In this study, we investigated the effect of either the simultaneous or staggered application of commercially available phages and ciprofloxacin versus *S. aureus/P. aeruginosa* dual-species biofilms *in vitro*. Biofilms were grown on porous glass beads and analyzed over time. Different techniques such as microcalorimetry, sonication and scanning electron microscopy were combined for the evaluation of anti-biofilm activities. Both bacterial species were susceptible to ciprofloxacin and to phages in their planktonic form of growth. Ciprofloxacin tested alone against biofilms required high concentration ranging from 256 to >512 mg/L to show an inhibitory effect, whereas phages alone showed good and moderate activity against MRSA biofilms and dual-species biofilms, respectively, but low activity against *P. aeruginosa* biofilms. The combination of ciprofloxacin with phages showed a remarkable improvement in the anti-biofilm activity of both antimicrobials with complete eradication of dual-species biofilms after staggered exposure to Pyophage or Pyophage + Staphylococcal phage for 12 h followed by 1 mg/L of ciprofloxacin, a dose achievable by intravenous or oral antibiotic administration. Our study provides also valuable data regarding not only dosage but also an optimal time of antimicrobial exposure, which is crucial in the implementation of combined therapies.

## Introduction

Although many common infectious diseases can be initiated by a single pathogen or virulence factor, others can be attributed to a polymicrobial origin (Peters et al., 2012). *Staphylococcus aureus* and *Pseudomonas aeruginosa* are commonly found in mixed biofilm infections including chronically infected wounds, indwelling medical devices, cystic fibrosis lung infection or diabetic foot ulcers among others (Tande and Patel, 2014; Chew et al., 2018). Usually, polymicrobial biofilm infections result in worse clinical outcomes than the single infections caused by either species (Serra et al., 2015; Limoli et al., 2016). Treatment is often complicated due to the synergies of polymicrobial biofilms on limiting the effectiveness of antibiotics (Wolcott et al., 2013). Radlinski found that the interaction of *S. aureus* with *P. aeruginosa* within a biofilm can alter *S. aureus*’ susceptibility to different antibiotics (Radlinski et al., 2017), whereas other authors also suggested a phenotypic change of *S. aureus* to a small colony variant (SCV) in the presence of *P. aeruginosa* (Chew et al., 2018), increasing its tolerance toward antibiotics (Garcia et al., 2013).

The lack of effective therapies against polymicrobial biofilm infections is a pressing need for the development of new antimicrobial strategies. Bacteriophages (phages) have regained interest as promising therapeutic option in fighting biofilm-related infections due to their rapid bactericidal activity, the self-amplification ability and potential biofilm degradative properties (Harper et al., 2014). However, there are only limited studies investigating the activity of phages against polymicrobial biofilms (Sillankorva et al., 2010; Kay et al., 2011; Chhibber et al., 2015; Oliveira et al., 2018; Melo et al., 2019) and just recently Akturk et al. (2019) evaluated the simultaneous and staggered administration of a *P. aeruginosa*-targeting monophage and conventional antibiotics on *S. aureus/P. aeruginosa* dual-species biofilms.

Pyophage (PYO) and Staphylococcal bacteriophage (Sb-1) are two commercially available phage preparations manufactured by Eliava Biopreparations, a company associated with the G. Eliava Institute of Bacteriophages, Microbiology and Virology, Tbilisi, Georgia. Sb-1 is a *Staphylococcus*-targeting phage preparation containing the well characterized and fully sequenced Sb1 phage (Kvachadze et al., 2011), whereas PYO is composed by a cocktail of phages targeting *S. aureus, Streptococcus spp.*, Escherichia *coli, P. aeruginosa*, and *Proteus species*. An advantage of using phages cocktails lies in a more broad antibacterial spectrum of activity while minimizing the emergency of bacterial resistance (Chan et al., 2013). Furthermore, phages may encode extracellular polysaccharides (EPS) depolymerases to facilitate their penetration within biofilms (Fernandes and Sao-Jose, 2018). Indeed, in a previous study, we observed the ability of Sb-1 to degrade the extracellular polysaccharide component of *S. aureus* biofilm, which could have improved synergism with antibiotics (Tkhilaishvili et al., 2018b). Thus, in the present study, we investigated the effectiveness of both phage preparations to enhance antibiotic activity in eradicating *S. aureus/P. aeruginosa* dual-species biofilm. We hypothesize that while PYO can target both bacterial species, the addition of Sb-1 targeting not only *S. aureus* but also the biofilm matrix, could help in completely eradicating the dual-species biofilm when combined with an antibiotic. Mono- and dual-species biofilms of *S. aureus* and *P. aeruginosa* were reproducibly grown in porous glass beads and exposed to phages, ciprofloxacin and their simultaneous or staggered combinations. Furthermore, the morphological changes of biofilms induced by each treatment condition were analyzed with scanning electron microscopy (SEM).

## Materials and Methods

### Bacterial Strains and Bacteriophages

Methicillin-resistant *S. aureus* (MRSA) ATCC 43300 and *P. aeruginosa* ATCC 27853 strains were used in this study. Bacteria were stored on a cryovial bead preservation system (Microbank; Pro-Lab Diagnostics, ON, Canada) at −80 °C.

Phages Sb-1 and PYO were provided as 10 mL liquid ampoules by the Eliava Institute for Bacteriophages, Microbiology and Virology (Tbilisi, Georgia) and maintained at 4°C. The phage titer was determined by titration and expressed as PFU/mL. A fixed titer of PYO corresponding to 10^5^ PFU/mL for MRSA and 10^4^ PFU/mL for *P. aeruginosa* and of Sb-1 corresponding to 10^6^ PFU/mL for MRSA were used for all tests.

### Biofilm Formation Assay

In this study, we applied an optimized *in vitro* assay for biofilm formation using porous sintered glass beads (diameter, 4 mm; pore size, 60 μm; porosity, 0.2 m^2^/g; ROBUVR, Hattert, Germany) following the assay described by Zimmerli et al. (1994) with some modifications.

Considering the findings from previous studies predominantly showing an out-competition of *S. aureus* growth by *P. aeruginosa* growth (Filkins et al., 2015; Woods et al., 2019) bacterial inoculums in our study were prepared at a ratio of 1 *P. aeruginosa* to 1000 MRSA bacterial cells.

In order to allow mono- and dual-species biofilm formation on the glass beads, a bacterial suspension of MRSA corresponding to 5 × 10^6^ CFU/mL and *P. aeruginosa* corresponding to 5 × 10^3^ CFU/mL were incubated – alone or combined – in Luria-Bertani broth (LB, Sigma-Aldrich, Steinheim, Germany) in the presence of porous glass beads at 37°C under static conditions. After 3, 6, 12, or 24 h of incubation, beads were washed three times in sterile 0.9% saline to remove non-adherent bacteria suspended in the incubation medium. The number of MRSA and *P. aeruginosa* bacteria adhering on the glass beads was determined by sonication and colony counting (see section “Sonication of Biofilms Formed on Porous Glass Beads and Plating for Colony Counting”) in Mannitol salt agar (VWR Chemicals, Leuven, Belgium) and Cetrimide selective agar media (Sigma-Aldrich, Steinheim, Germany) respectively. The dual-species biofilm formed in the beads was also visualized by SEM (see section “SEM of Biofilms on Porous Glass Beads”).

Twenty-four hours old dual-species biofilms with a 1:1 ratio of MRSA and *P. aeruginosa* bacterial cells on the beads were used for anti-biofilm activity tests.

### Sonication of Biofilms Formed on Porous Glass Beads and Plating for Colony Counting

The presence of attached cells to the glass beads was evaluated by CFUs counting of sonicated beads as previously described (Gonzalez Moreno et al., 2019). After biofilm formation, glass beads were washed three times with 0.9% saline and introduced in Eppendorf tubes containing 1 mL of sodium-phosphate buffer solution (PBS). Samples were vortexed for 30 s and then subjected to sonication in an ultrasound bath at 40 kHz and 0.2 W/cm^2^ (BactoSonic, BANDELIN electronic GmbH & Co., KG, Berlin, Germany) for 1 minute, followed by additional 30 s vortexing. 10-fold serial dilutions of the sonication fluid were plated onto the appropriate media and colonies were counted after 18–24 h incubation at 37°C and expressed as CFUs/mL.

### SEM of Biofilms on Porous Glass Beads

For SEM imaging, biofilm was formed on porous glass beads as described above. Afterward, all beads were washed in ddH_2_O (dipping) to remove unbound bacteria and chemically fixed. Subsequently, the samples were dehydrated in ethanol percent series and then dried at the critical point. Samples were mounted on aluminum stubs, coated with 20 nm layer of gold-palladium, and then observed in the microscope (DSM 982 GEMINI, Zeiss Oberkochen).

### Antimicrobial Assay by Microcalorimetry and Sonication/Colony Counting

An isothermal microcalorimeter (TAM III; TA Instruments, New Castle, DE, United States) equipped with 48 channels was used to determine the antimicrobial activity of either antibiotic and/or phages against planktonic, mono- and dual-species biofilms as previously reported (Butini et al., 2018; Tkhilaishvili et al., 2018a, b). Briefly, MRSA or *P. aeruginosa* planktonic cells (10^5^ CFU/mL) were exposed to two-fold serial dilutions of ciprofloxacin or to each phage preparation in LB, and heat production was measured for 24 h. The minimum heat inhibitory concentration (MHIC) was defined as the lowest concentration of antimicrobial able to suppress the metabolic heat production of planktonic bacteria.

Mono- and dual-species biofilms formed on porous glass beads as previously described were rinsed (3×) with 0.9% saline and exposed to fresh LB containing ciprofloxacin or phages. After 24 h of incubation at 37°C, beads were rinsed (3×) with 0.9% saline and inserted in microcalorimetry ampoules containing 3 mL of fresh LB and introduced into the calorimeter. The viability of bacteria on the glass beads after the antibiotic treatment was detected by measuring their heat production at 37°C for 48 h. For samples where not heat production was detected, the complete biofilm eradication was determined by CFU counting of the sonicated beads after the microcalorimetric assay. The minimum biofilm bactericidal concentration (MBBC) was defined as the lowest concentration of antibiotic that strongly reduced the viability of biofilm cells and led to the absence of heat flow production from treated beads when incubated during 48 h in fresh medium. The minimum biofilm eradicating concentration (MBEC) was defined as the lowest concentration of antibiotic required to kill all sessile cells resulting in the appearance of no colony after plating sonication fluid (detection limit: 20 CFU/mL) (Gonzalez Moreno et al., 2019; Wang et al., 2019). All experiments were performed in triplicate and repeated three times.

To evaluate the antimicrobial effect of antibiotic/phage combinations, two different approaches were carried out: (i) simultaneous exposure of biofilms to PYO or PYO+Sb-1 and sub-inhibitory concentrations of ciprofloxacin for 24 h; (ii) staggered exposure of biofilms to PYO or PYO+Sb-1 phages for 3, 6, 12, or 24 h followed by a 24 h-exposure to sub-inhibitory concentrations of ciprofloxacin. The viability of bacteria on the glass beads after the antibiotic/phage treatment was determined by both, calorimetry and sonication/colony-counting as previously mentioned.

For each tested condition throughout all experiments, grown biofilms were rinsed with 0.9% saline prior exposure to fresh LB containing the respective antimicrobials.

Microcalorimetry data was evaluated using the manufacturer’s software (TAM Assistant; TA Instruments, New Castle, DE, United States) and figures were plotted using GraphPad Prism 6.01 (GraphPad Software, La Jolla, CA, United States).

## Results

### Formation of Mono- and Dual-Species Biofilm

**FIGURE 1:**
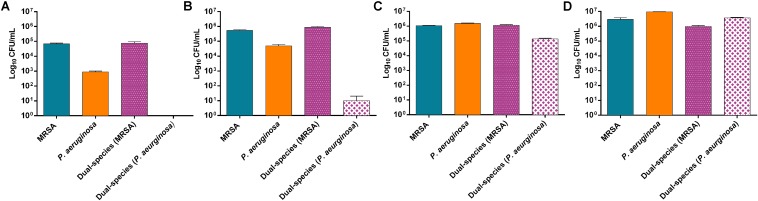
Distribution pattern of bacterial populations over the time. Number of viable cells (in log10 CFU/mL) of *P. aeruginosa* and MRSA on mono- and dual-species biofilms formed after 3 h **(A)**, 6 h **(B)**, 12 h **(C)** and 24 h **(D)**. Data are reported as CFU/mL mean ± standard deviation of at least three independent experiments. Figure 2 | Dual-species biofilm formed by MRSA (ATCC 43300) and *P. aeruginosa* (ATCC 27853) on porous glass beads after 24 h of incubation. Image **(B)** is a close-up from **(A)**. Numbers 1 and 2 indicate a MRSA bacterium and a *P. aeruginosa* bacterium, respectively, whereas three and four point out a water channel and the extracellular polymeric matrix of the biofilm correspondingly.

MRSA and *P. aeruginosa* were used to grow mono- and dual-species biofilms. The evaluation of the bacteria adhered to the beads over time showed a considerably higher concentration of MRSA cells at 3 and 6 h of incubation in dual-species biofilms compared to *P. aeruginosa* cells (Figure 1A,B), whereas at 12 h of incubation the concentration of *P. aeruginosa* increased substantially (Figure1C) and at 24 h of incubation the concentration of *P. aeruginosa* showed values comparable to those from MRSA (Figure 1D).

Results showed approximately a 1:1 ratio of MRSA (9.7 × 10^5^ CFU/mL) and *P. aeruginosa* (3.7 × 10^6^ CFU/mL) bacterial cells on the beads of dual-species biofilms after 24 h of incubation. MRSA and *P. aeruginosa* mono-species biofilms presented bacterial concentrations comparable to those observed on the dual-species biofilm after 24 h of incubation.

**FIGURE 2:**
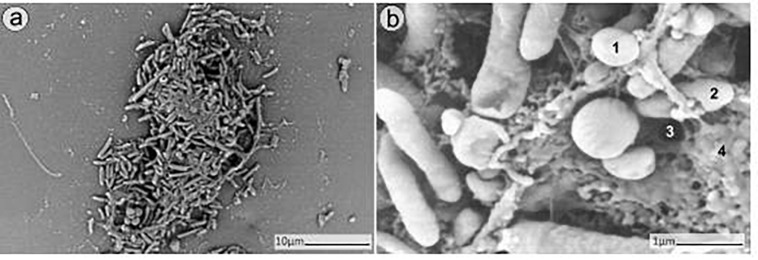
SEM analysis of dual-species biofilm formed by MRSA (ATCC 43300) and *P. aeruginosa* (ATCC 27853) on porous glass beads after 24h of incubation. Image **(b)** is a close-up from **(a)**. Numbers 1 and 2 indicate a MRSA bacterium and a *P. aeruginosa* bacterium respectively, whereas 3 and 4 point out a water channel and the extracellular polymeric matrix of the biofilm correspondingly.

The SEM analysis of 24 h-old dual-species biofilms showed the capability of MRSA and *P. aeruginosa* to adhere and form an even mixed biofilm on the porous glass beads (Figure 2).

### Antimicrobial Activity of Ciprofloxacin or Phages Against Planktonic, Mono- and Dual-Species Biofilms

**FIGURE 3:**
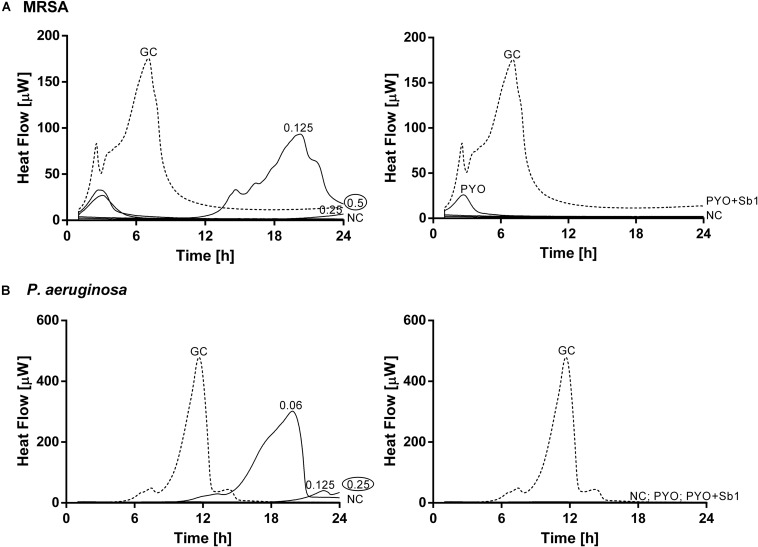
Microcalorimetry analysis of planktonic MRSA ATCC 43300 **(A)** and *P. aeruginosa* ATCC 27853 **(B)** cells treated with two-fold increasing concentrations of ciprofloxacin (left column, numbers represent concentrations in mg/L of antibiotic) or with phages (right column). A circled value represents the MHIC, defined as the lowest concentration of antimicrobial able to suppress the metabolic heat production of planktonic bacteria. GC, growth control (dashed line); NC, negative control. Data of a representative experiment are reported.

**FIGURE 4:**
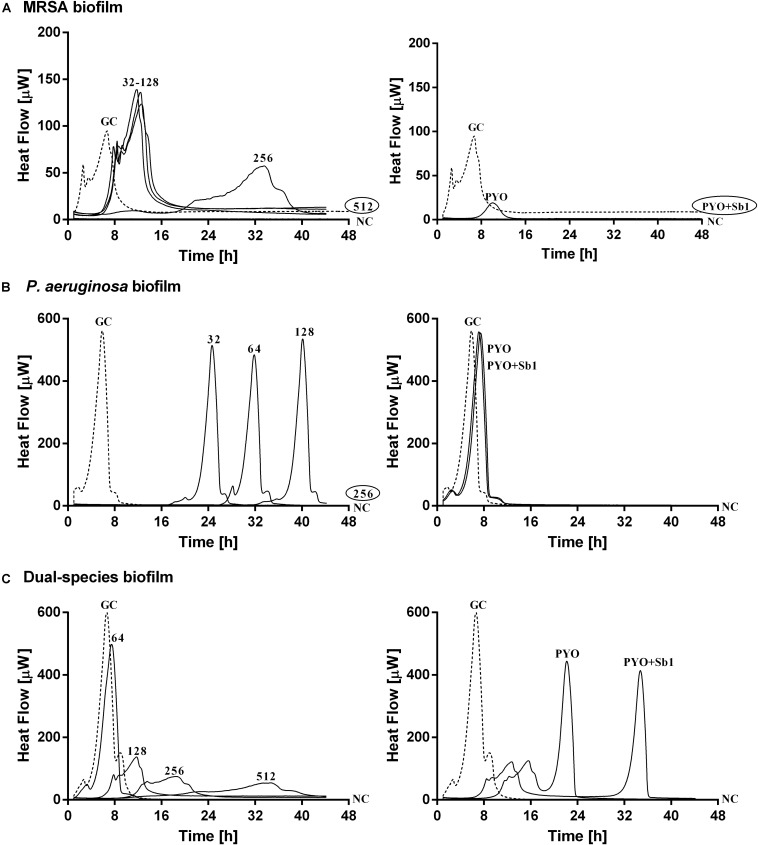
Microcalorimetry analysis of **(A,B)** mono- and **(C)** dual-species MRSA (ATCC 43300) and *P. aeruginosa* (ATCC 27853) biofilms treated with two-fold increasing concentrations of ciprofloxacin (left column, numbers represent concentrations in mg/L of antibiotic) or with phages (right column). Each curve shows the heat produced by viable bacteria present in the biofilm after 24 h of antibiotic or phage treatment. A circled value represents the MBEC, defined as the lowest concentration of antibiotic that strongly reduced the viability of biofilm cells leading to the absence of heat flow production from treated beads when incubated during 48 h in fresh medium and no colonies after sonication and plating. GC, growth control (dashed line); NC, negative control. Data of a representative experiment are reported.

The antimicrobial susceptibility of planktonic cells (Figure 3) or mono- and dual-species biofilms (Figure 4) to ciprofloxacin or to phages was determined by isothermal microcalorimetry.

The calorimetry analysis for planktonic bacteria showed that MRSA and *P. aeruginosa* were susceptible to ciprofloxacin with MHIC values of 0.5 and 0.25 mg/L, respectively. The exposure of MRSA to PYO revealed a high reduction of heat production compared to the growth control, a complete inhibition of the heat production could be observed in the case of *P. aeruginosa*. The combination of PYO+Sb-1 showed complete growth inhibition against both bacterial species.

Mono-species biofilms from both strains were susceptible to considerable high concentrations of ciprofloxacin (512 mg/L for MRSA and 256 mg/L for *P. aeruginosa*), whereas the antibiotic was not able to completely inhibit the heat flow production of the dual-species biofilm when tested up to 512 mg/L.

The exposure of MRSA biofilm to PYO revealed a drastic reduction of the heat production compared to the growth control, and with the addition of Sb-1, a complete inhibition of the biofilm could be achieved. On the contrary, neither PYO nor PYO+Sb1 showed a noteworthy anti-biofilm activity against *P. aeruginosa* biofilm, whereas on dual-species biofilm, a delay on the heat production could be observed on treated samples with PYO, indicating a moderate anti-biofilm activity, which was seen improved by the addition of Sb1 but with no complete inhibition of the biofilm.

### Biofilm-Eradicating Activity of Phage Preparations

In order to evaluate the biofilm-eradicating activity of the two phage preparations, mono- and dual-species biofilms were exposed to PYO or to PYO+Sb-1 for 24 h and then, viable bacteria attached to the beads were detected by colony counting after bead sonication and plating of the sonication fluids.

**FIGURE 5:**
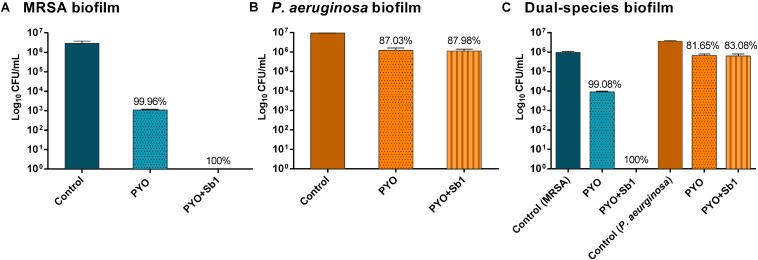
Effect of PYO and Sb-1 phage preparations on viability of biofilm-embedded mono and mix bacteria populations. *S. aureus/P. aeruginosa* mono- **(A,B)** and dual-species **(C)** biofilms formed on porous glass beads were exposed to phages. Data are reported as log10 CFUs/mL mean ± standard deviation of at least three independent experiments. Percent of cell reduction of treated samples compared to untreated samples was calculated as: percent reduction = [(A–B)/A]×100, where A is the mean number of viable bacteria of the growth control and B is the mean number of viable bacteria after exposure to PYO or PYO+Sb-1.

A higher reduction of MRSA viable bacteria after exposure to PYO could be observed (Figure 5A) compared to *P. aeruginosa* biofilm, where no considerable bacterial reduction was determined (Figure 5B). Moreover, a complete eradication of MRSA biofilm was observed after exposure to PYO+Sb-1, although this phage combination did not improve the killing of *P. aeruginosa* biofilm compared to PYO alone.

Regarding the phage activity against dual-species biofilm (Figure 5C), a reduction of more than 2 log10 of MRSA and around 1 log10 *of P. aeruginosa* cells was observed after exposure to PYO in comparison to the growth control. The combination of PYO+Sb-1 showed a complete eradication of MRSA cells on the dual-species biofilm, whereas no substantial reduction of *P. aeruginosa* cells was observed.

### Anti-biofilm Activity of Ciprofloxacin in Combination With Phages Against Dual-Species Biofilm

**FIGURE 6:**
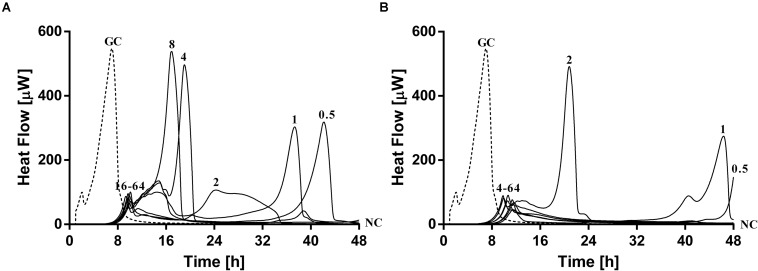
Evaluation of MRSA ATCC 43300/*P. aeruginosa* ATCC 27853 dual-species biofilm viability after simultaneous exposure during 24 h to ciprofloxacin at increasing doses (0.5–64 mg/L) plus **(A)** PYO or **(B)** PYO+Sb-1 monitored by microcalorimetry. Numbers represent antibiotic concentrations (in mg/L). GC, growth control (dashed line); NC, negative control.

Simultaneous exposure of sub-inhibitory concentrations of ciprofloxacin combined with PYO or PYO+Sb-1 revealed a remarkable delay and reduction of heat flow production compared to the heat flow produced by the growth control (Figure 6).

The combination of PYO and ciprofloxacin revealed a decrease of over 90% in heat flow production of samples treated with 16–64 mg/L of antibiotic compared to the growth control, whereas in the case of combining PYO+Sb-1 and ciprofloxacin, a concentration of antibiotic as low as 4 mg/L was already able to reduce over a 90% of the heat flow production if compared to that one measured for the growth control. Still, no complete biofilm eradication was observed with any of the both tested treatment combinations.

Paradoxically, low concentrations of ciprofloxacin (0.5–1 mg/L) in combination with phages showed a higher delay/decrease in heat flow production, correlating with a lower loading of bacterial cells in the beads, if compared to the heat flow curves observed after exposure to concentrations of ciprofloxacin ranging from 2 to 8 mg/L, in combination with phages.

**FIGURE 7:**
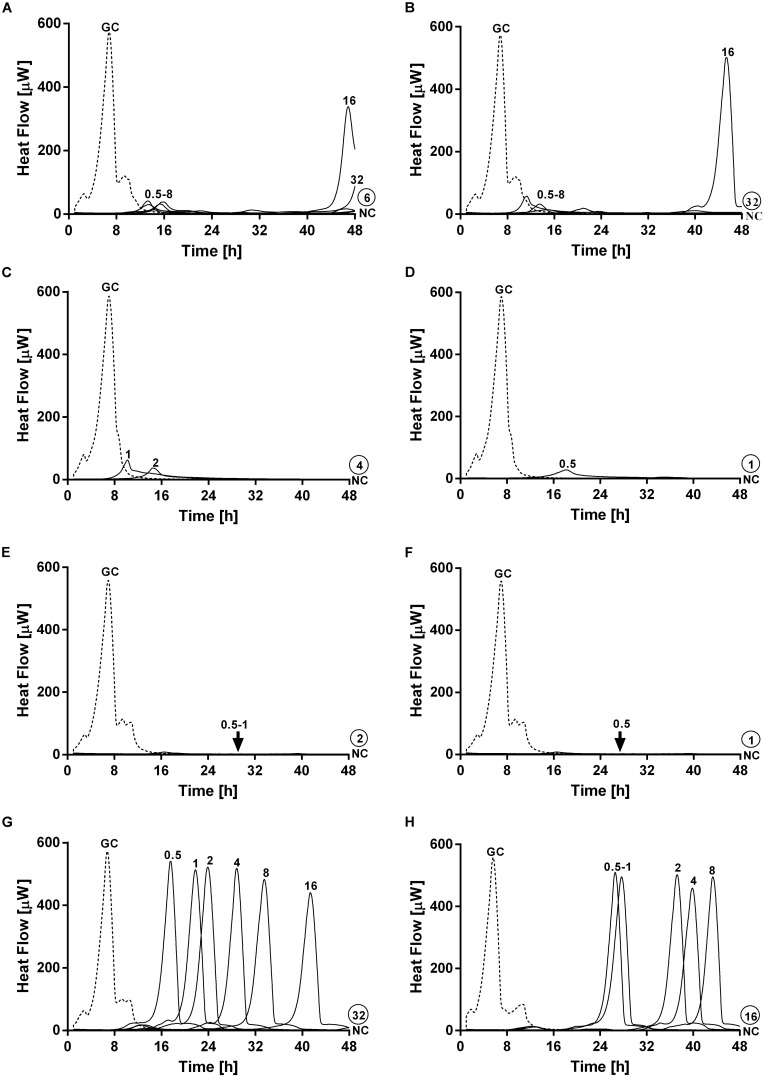
Evaluation of MRSA ATCC 43300/*P. aeruginosa* ATCC 27853 dual-species biofilm viability after staggered exposure to phages and ciprofloxacin monitored by microcalorimetry. Each curve shows the heat produced by viable bacteria present in biofilms pretreated for 3 h **(A,B)**, 6 h **(C,D)**, 12 h **(E,F)** and 24 h **(G,H)** with PYO (graphs on the left) or PYO+Sb-1 (graphs on the right) followed by exposure to ciprofloxacin at increasing doses (0.5–64 mg/L) for 24 h. Numbers above curves represent antibiotic concentrations (in mg/L). Circled values represents the MBEC, defined as the lowest concentration of antibiotic that strongly reduced the viability of biofilm cells leading to the absence of heat flow production from treated beads when incubated during 48 h in fresh medium and no colonies after sonication and plating. GC, growth control (dashed line); NC, negative control.

On the other hand, a staggered exposure of phage and antibiotic against dual-species biofilms was evaluated by microcalorimetry. The obtained results are depicted in Figure 7. Dual-species biofilms were first exposed to phages at different incubation times (3, 6, 12, and 24 h) and then to sub-inhibitory concentrations of ciprofloxacin for 24 h. After calorimetry, the complete eradication of the biofilm was further investigated by sonication and colony counting of those samples showing no heat flow production after 48 h of incubation.

Results showed the highest anti-biofilm activity when the antibiotic was added after 12 h of pre-exposure to either PYO or PYO+Sb-1, where a complete eradication of the biofilm could be achieved at MBEC of ciprofloxacin of 2 mg/L (Figure 7E) and 1 mg/L (Figure 7F) respectively. Similarly, relatively low MBEC values were also obtained when ciprofloxacin was added after 6 h of biofilm pre-treatment with PYO (MBEC = 4 mg/L) (Figure 7C) or PYO+Sb-1 (MBEC = 1 mg/L) (Figure 7D). On the contrary, when biofilms were incubated for 3 or 24 h with phages prior addition of ciprofloxacin, higher MBEC values ranging from 16 to 64 mg/L were observed. Generally, the PYO+Sb-1/ciprofloxacin combination exhibited MBEC values 2–4 times lower than the PYO/ciprofloxacin combination at all the tested incubation times.

### SEM Analysis

**FIGURE 8:**
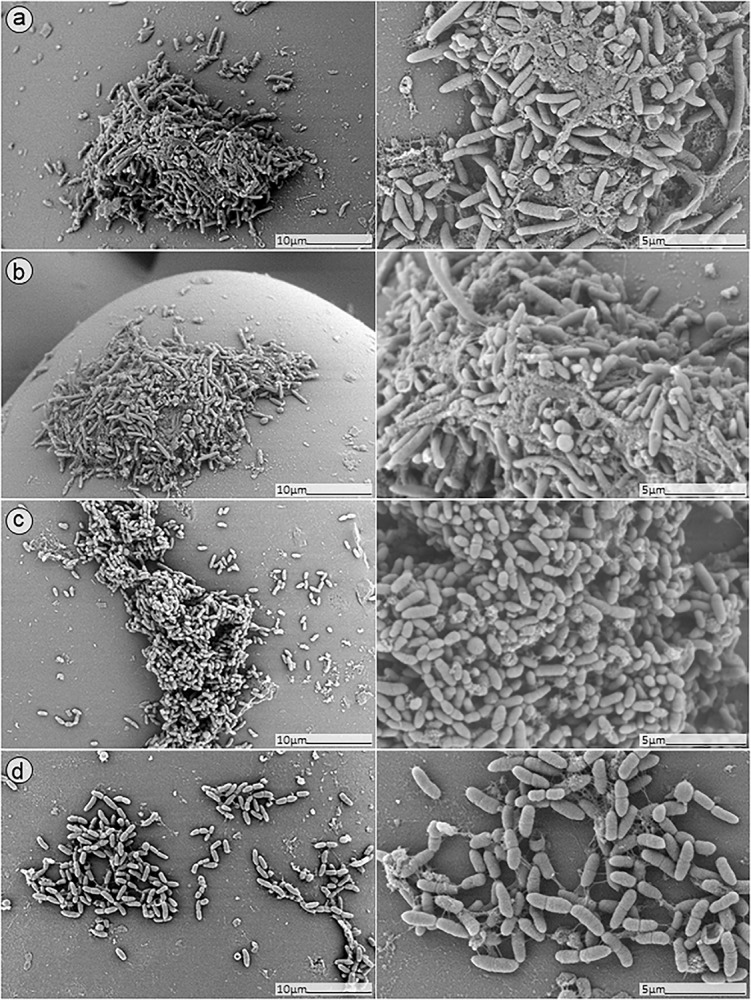
SEM analysis of *S. aureus/P. aeruginosa* dual-species biofilms grown on porous glass beads for 24 h without treatment **(a)** and after exposure to 24 h monotherapy with **(b)** ciprofloxacin (1 mg/L); **(c)** PYO; or **(d)** pyo+sb-1.

**FIGURE 9:**
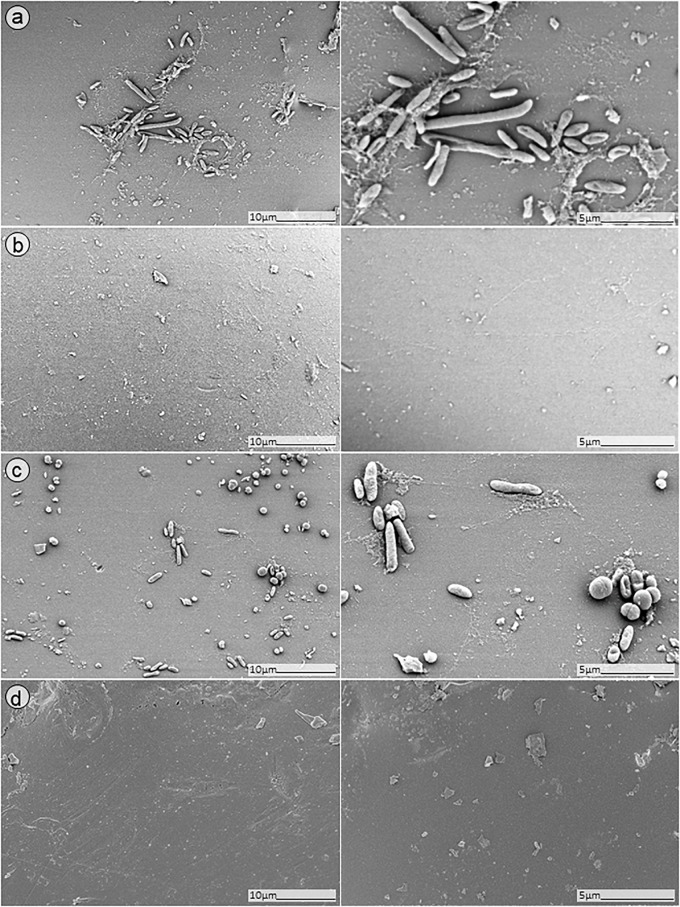
SEM analysis of *S. aureus/P. aeruginosa* dual-species biofilm grown on porous glass beads for 24 h and treated with a combinatorial therapy of **(a)** simultaneous exposure to PYO and ciprofloxacin (1 mg/L, 24 h); **(b)** staggered exposure to PYO (12 h) followed by ciprofloxacin (1 mg/L, 24 h); **(c)** simultaneous exposure to PYO+Sb-1 and ciprofloxacin (1 mg/L, 24 h); or **(d)** staggered exposure to PYO+Sb-1 (12 h) followed by ciprofloxacin (1 mg/L, 24 h).

In order to further confirm our findings, dual-species biofilm after exposure to either antimicrobials alone or in combinations were visualized by SEM (Figures 8, 9). The microscopy analysis revealed comparable outcomes to those obtained by microcalorimetry and sonication/colony-counting.

No presence of bacteria on the beads was observed after biofilm exposure to phages for 12 h followed by 24 h of exposure to 1 mg/L ciprofloxacin (Figure 9, images B,D). Ciprofloxacin alone at that same concentration (1 mg/L) showed abundant biofilm formation on the bead (Figure 8, image B), indicating no anti-biofilm activity at that concentration.

Different outcomes were observed when biofilms were exposed to PYO+Sb-1 (Figure 8D), were a relatively lower abundance of biofilm and especially of MRSA bacterium cells could be visualized, compared to PYO alone (Figure 8C). Finally, the beads exposed to simultaneous incubation with phages and ciprofloxacin (Figure 9, images A,C) showed a sharp decrease of biofilm without complete eradication.

## Discussion

*S. aureus* and *P. aeruginosa* are two bacterial pathogens commonly isolated in mixed-species biofilm infections (Hotterbeekx et al., 2017). A vast number of studies suggest that, when both bacterial species interact to form biofilm, *S. aureus* is predominantly outcompeted by *P. aeruginosa* (Machan et al., 1991; Mashburn et al., 2005; Filkins et al., 2015; Woods et al., 2019). However, other studies found that both species may benefit each other during the infection and keep a stable co-existence (Pastar et al., 2013; DeLeon et al., 2014; Woods et al., 2019). In our study, we observed a similar trend, where the growth of *S. aureus* biofilm was outcompeted by *P. aeruginosa* during a period of 24 h *in vitro*, despite the higher initial inoculum size of *S. aureus* in relation to *P. aeruginosa*, and both species could form an even mixed biofilm after 24 h of co-incubation, as shown by colony-counts and SEM.

It has been shown that *S. aureus/P. aeruginosa* coinfections result in enhanced virulence and resistance to antibiotics (DeLeon et al., 2014). Our results also revealed that a higher concentration of ciprofloxacin was necessary to inhibit the growth of dual-species biofilms when compared with mono-species biofilms. The MBEC values obtained in all cases are too high to be reached in the clinical practice (Kontou et al., 2011; Thabit et al., 2019). In this scenario, bacteriophages appear to be an alternative strategy to treat biofilm-forming infections. Over the past few years, numerous studies have been carried out investigating the effectiveness of phages against mono- and dual-species biofilm (Sillankorva et al., 2010; Chhibber et al., 2015; Gutierrez et al., 2015; Gonzalez et al., 2017; Melo et al., 2019). Many of these studies pointed to a notable dependency between the phages and the bacterial species involved on the biofilm for the efficacy of the phage treatment. Indeed, it is generally accepted that the efficacy of phages against bacteria is influenced by several factors, among others, the host specificity, the treatment method, environmental conditions or accessibility to target bacteria (Ly-Chatain, 2014). The impact of host specificity for the therapeutic use of phages is also under debate (Ross et al., 2016; Hyman, 2019). Thus, in contrast to other studies where they make use of self-isolated phages targeting the bacterial strains under study, for our study we chose to investigate commercially available phage preparations with the potential to a more straightforward implementation in a clinical setting.

Our results showed that, even though planktonic cells from both tested species were susceptible to the PYO phage-cocktail, when tested against biofilms, only MRSA showed a substantial reduction on bacterial viability, especially as mono-species biofilm, whereas a lower efficacy was observed against dual-species biofilms. A possible explanation for this might be a limited phage penetration within the biofilm, what could be improved by the addition of the MRSA targeting and matrix-degrading Sb-1 phage. The combination of PYO+Sb-1 showed a major eradication of MRSA cells on the dual-species biofilm, as seen also by SEM, however, no substantial reduction of *P. aeruginosa* cells was observed. A possible additional effect on the reduction of MRSA could be due to the natural competition between the two species as mentioned above.

To enhance the effect of phages, the combined exposure with an antibiotic was assessed. Phage- antibiotic synergy is the result of combining sub-inhibitory concentrations of antibiotics with phages to foster phage productivity and thus phage-mediated bacterial decline (Tagliaferri et al., 2019). Previous studies have shown the benefit of the staggered application when combining antibiotics and phages, while a simultaneous exposure could result in hindering their anti-biofilm efficacy, possibly due to antagonistic modes of action (Chaudhry et al., 2017; Kumaran et al., 2018; Akturk et al., 2019). Indeed, when we analyzed by calorimetry the exposure of dual-species biofilms to ciprofloxacin and PYO or PYO+Sb-1 simultaneously, we observed a paradoxical effect, where lower concentrations of ciprofloxacin in combination with phages showed a higher delay/decrease in heat flow production compared to higher antibiotic concentrations. We assume that the mode of action of ciprofloxacin inhibiting bacterial DNA replication might hamper the phage amplification (replication) (Constantinou et al., 1986). Therefore, lower doses of ciprofloxacin could have a minor interference with phage replication or could not reduce the concentration of bacteria to levels below which phages can replicate, if compared to higher antibiotic doses (Levin et al., 1977). This counterproductive effect could be perhaps prevented by the use of antibiotics with modes of action that do not compete with the viral amplification, or also, by exposing bacteria in a staggered rather than a simultaneous manner to phages and antibiotics. As seen in our study, a complete eradication of dual-species biofilm could be only achieved by staggered administration of phages followed by a sub-inhibitory concentration of ciprofloxacin.

As recently stated by Tagliaferri et al. (2019), synergistic interactions between antimicrobial agents may be strongly dependent on the treatment conditions such as dosage, frequency, time points and order of administration. Hence, we were interested on determining the optimal time point for the staggered administration of phages and the antibiotic. Our results showed that the highest anti-biofilm activity could be reached when ciprofloxacin was added after 6 or 12 h of pre-exposure to PYO+Sb-1. SEM analysis also revealed the absence of adherent bacterial cells on the glass beads.

Differently, pre-incubation of dual-species biofilms with phages for 3 or 24 h prior addition of ciprofloxacin exhibited higher MBEC values, confirming that, not only dosage but also an optimal time of antimicrobial exposure is crucial in the implementation of the combined therapies.

In conclusion, this work provides valuable original data on the combinatorial use of phage and antibiotic against *S. aureus/P.aeruginosa* dual-species biofilm that might bring new insights into the potential application of such a treatment to combat polymicrobial infections. Monotherapy with ciprofloxacin revealed drug concentrations to eradicate biofilm (MBEC >512 mg/L) much superior to the ones reachable in clinical practice, whereas a combinatorial treatment by staggered administration of phages and ciprofloxacin strongly reduced the MBEC of ciprofloxacin to a dose (MBEC = 1 mg/L) achievable by intravenous or oral antibiotic administration (Kontou et al., 2011; Thabit et al., 2019). Moreover, by the use of commercially available phage preparation in this study, we were able to show the effectiveness of these preparations against bacterial strains that have not been used specifically for their isolation.

Over the last years, bacteriophages have been extensively studied as therapeutic agents alone or in conjunction with other therapeutics. *In vivo* models (Tagliaferri et al., 2019) and a few clinical trials (Merabishvili et al., 2017; Furfaro et al., 2018; Jault et al., 2019) have demonstrated effectiveness of phage treatment against *P. aeruginosa* and *S. aureus* infections, without any reported adverse effects. However, little has been published about polymicrobial biofilm infections. Although these infections are less common, their treatment presents a major challenge. Hence, further preclinical and clinical studies are essential to support the development of phage/antibiotic combination therapy for polymicrobial infections.

## Data Availability Statement

The raw data supporting the conclusions of this article will be made available by the authors, without undue reservation, to any qualified researcher.

## Author Contributions

TT and AT conceived and designed the experiments. TT performed the experiments. TT and MG analyzed the data and drafted the manuscript, with the contribution of LW, CP, and AT. All authors reviewed and revised the final drafts of this manuscript.

## Conflict of Interest

The authors declare that the research was conducted in the absence of any commercial or financial relationships that could be construed as a potential conflict of interest.
